# Design of Catalase Monolithic Tablets for Intestinal Targeted Delivery

**DOI:** 10.3390/pharmaceutics13010069

**Published:** 2021-01-07

**Authors:** Mirna Alothman, Pompilia Ispas-Szabo, Mircea Alexandru Mateescu

**Affiliations:** Department of Chemistry and Centre CERMO-FC, Research Chair in Prevention of Gastroenteric Dysfunctions “Allerdys”, Université du Québec à Montréal, C.P. 8888, Branch A, Montréal, QC H3C 3P8, Canada; alothman.mirna@courrier.uqam.ca (M.A.); mateescu.m-alexandru@uqam.ca (M.A.M.)

**Keywords:** catalase, therapeutic enzyme, ampholytic starch, intestinal inflammation, drug-targeted formulations, ionic matrices, hydrogen peroxide

## Abstract

Several studies confirmed a correlation between elevated hydrogen peroxide (H_2_O_2_) levels in patients with intestinal bowel diseases (IBD) and the negative effects caused by its presence. The objective of this study was to explore the potential use of catalase (CAT) to diminish the level of H_2_O_2_ and its deleterious action on intestinal mucosa. Oral dosage forms of a CAT bioactive agent targeted to the intestines were designed and tested in various simulated gastric and intestinal media. Monolithic tablets (30% loading) were prepared using commercial CarboxyMethylCellulose (CMC) or synthesized CarboxyMethylStarch (CMS) and TriMethylAmineCarboxyMethylStarch (TMACMS) as matrix-forming excipients. For starch derivatives, the presence of the ionic groups (carboxymethyl and trimethylamine) was validated by spectral analysis. In vitro studies have shown that tablets formulated with TMACMS and 30% CAT resisted the acidity of the simulated gastric fluid and gradually released the enzyme into the simulated intestinal fluid. The investigation of the CAT release mechanism revealed the role of anionic and cationic groups of polymeric excipients and their involvement in the modulation of the CAT dissolution profile. The proposed drug delivery system can be considered an efficient solution to target CAT release in the intestine and contribute to the reduction of H_2_O_2_ associated with intestinal inflammation.

## 1. Introduction

Inflammatory bowel diseases (IBD) are noninfectious heterogeneous intestinal dysfunctions, including ulcerative colitis (UC) and Crohn’s disease (CD), drastically affecting the intestinal mucosa. High levels of hydrogen peroxide are usually found in chronically inflamed tissues and are one of the factors involved in increasing the deterioration of the disease [1].

Numerous studies have revealed that the bacterial populations inhabiting the gut play a key role in causing and perpetuating gut inflammation [2,3]. One aspect is related to anaerobic enterobacteria (e.g., *Escherichia coli*) that will benefit from the nutritional and redox environment of the inflamed gut in detriment of a healthy gut microbiota [4]. The redox changes in the inflamed gut are related to increased oxygen levels [5] and, also, to the production of reactive oxygen species (ROS). These agents can contribute to tissue damage [6,7].

The H_2_O_2_ produced by commensal bacteria of the human gut may be involved in the pathomechanisms of IBD by perpetuating an inflammatory reaction and increasing apoptosis and necrosis. An in vitro study showed the effects of different concentrations of H_2_O_2_ on the apoptosis and necrosis of HT-29 line cells representing the human gut epithelium. The supernatant of the *Lactobacillus* strain producing hydrogen peroxide (*L. delbrueckii* CU/22) was able to induce both apoptosis and necrosis in cultured human epithelial HT-29 cells [1]. Regulatory mechanisms in eukaryotic cells involve gene transcription and the expression of genes encoding for antioxidative enzymes such as thioredoxin reductase, Mn^2+^-dependent superoxide dismutase (Mn-SOD), glutathione S-transferase (Ya subunit), and NADPH; quinone reductase are induced by oxidant stimuli. A eukaryotic transcription factor system specifically activated by peroxides is NF-KB (nuclear factor-kappa B). The H_2_O_2_ at micromolar concentrations (100–150 µM) can mobilize the sequestered cytoplasmic form of NF-KB in cultured cells [8] and, thus, facilitate interleukin 1 (IL-1) stimulation of the synthesis and the secretion of macrophage migration inhibitory factor (MIF) [9].

Furthermore, it was found that H_2_O_2_ contributes to motor dysfunction and to reducing the intracellular Ca^2+^ signal in UC. Since mechanisms of Ca^2+^ release and other contractile signal transduction pathways might be impaired in IBD, cytosolic Ca^2+^ levels have been investigated before and after the addition of catalase (CAT) to isolated circular muscle cells. The measurements showed a catalase-induced restoration of the Ca^2+^ level. Moreover, it was shown that H_2_O_2_ is also produced in the gut mucosa [10]. Reactive oxygen species (ROS), including H_2_O_2_, modulate intestinal epithelial ion transport, contributing to IBD-associated diarrhea [11].

Since the excess of H_2_O_2_ is deleterious for the intestine, its degradation could represent a possible approach to treat the mentioned dysfunctions.

Catalases are ubiquitous enzymes that are very efficient in degrading H_2_O_2_ to water and oxygen (2H_2_O_2_ → 2H_2_O + O_2_). Catalases in eukaryotes are tetramers, each subunit containing a ferric heme and a bound molecule of NADPH [12]. They are among the enzymes with the highest turnover rates known; under optimal conditions, each subunit can decompose 2 × 10^5^ mol of hydrogen peroxide per second. Less human CAT activity was noted in gastric adenocarcinoma [13] and in CD, where patients showed a permanent suppression of CAT activity in their mononuclear cells [14]. From these studies, it seems that increasing the CAT level may be a promising therapeutic approach to treat intestinal dysfunctions caused by the excess of H_2_O_2_.

The oral administration of CAT as a therapeutic enzyme for intestinal diseases is limited by some physiological parameters (i.e., biochemical degradation due to the highly acidic stomach secretions). Despite the fact that the biopharmaceutical field has exhibited a rapid increase in the last decade, the topic of formulations for the oral administration of enzymes was not frequently addressed. The great majority of studies targeted pancreatic enzymes [15]. Others used different formulations, such as mesoporous silica microparticles (MSP) for the active phenylalanine ammonia-lyase (PAL) [16]. There are also some engineering strategies for orally administrable enzymes to enhance their stability by functionalization (i.e., modification with polymers and manipulation of the enzyme structure) [17,18]. The enteric coatings are the most common approach to protect the enzymes for oral administration. They may be phthalates i.e., cellulose acetate phthalate (CAP), hydroxypropyl methylcellulose phthalate (HPMCP), or methacrylic acid copolymers (Eudragit^TM^). Both categories are synthetic materials with some toxicological limitations for phthalates [19]. Coating applications on enzyme-based dosage forms represent a very challenging process due to some parameters (i.e., temperature and solvents) that can drastically affect the enzyme activity and stability in time. Other approaches consist of the usage of capsules, and there are a few commercial food integrators (supplements) of catalase in the form of capsules advertised as a treatment for “grey hair”, but there are no studies proving the effectiveness of cellulose capsules in protecting against stomach acidity. None of catalase existing products address any of its effects on the inflammatory intestinal conditions mentioned before.

Therefore, gastroprotective matrices based on pH-responsive polymers appear to be the key elements for the oral drug delivery of bioactive agents to the intestinal tract. These polymers should exhibit two main features: (i) be able to protect the active agent against stomach acidity and (ii) to release it at the targeted site in a controlled manner to ensure the availability of the enzyme along the intestines [20]. Very few studies have shown the effectiveness of enzyme therapy with new formulations that do not require an enteric coating to protect the enzymes. CAT was formulated with diamine oxidase as monolithic tablets using carboxymethyl starch (CMS): chitosan biopolymers [21]. Previous studies of our group dedicated to the design of oral forms of bioactive agents such as *E. coli* bacteria [22], the Fimbriae F4 vaccine peptide [23], or small active pharmaceutical ingredients (APIs) [24,25] have shown that excipients carrying ionic groups were good matrix-forming materials. Different to these previous studies, the design of a new drug delivery system to successfully release CAT at the site of intestinal action is very challenging due to aspects related to the preservation of its conformation and activity.

Our approach consists in the selection of polymeric excipients that will not only protect, but will also control, the release of the bioactive agent in small amounts at predefined controlled rates [26,27]. The aim of this study was to investigate the features of different oral formulations based on novel or commercial excipients and their capacity to protect and deliver CAT to the gut with the perspective to reduce H_2_O_2_ and, possibly, to alleviate the intestinal inflammation. If two decades before the oral administration of enzymes was mostly indicated for lactose intolerance or for exocrine pancreatic insufficiency, more recently, new needs have appeared in relation with inflammatory diseases of the gut that are becoming growing health concerns.

## 2. Materials and Methods

### 2.1. Materials

Catalase from *Aspergillus niger* supplied by Parchem Fine & Specialty Chemicals (New Rochelle, NY, USA) was used without further purification. High amylose starch (Hylon VII) was a gift from Ingredion (Westchester, IL, USA). Sodium monochloroacetate (SMCA) and hydrogen peroxide were from Sigma-Aldrich Canada (Oakville, ON, Canada). Glycidyltrimethylammonium chloride (GTMAC) and sodium carboxymethylcellulose (CMC) were purchased from Sigma-Aldrich Canada (Oakville, ON, Canada). Pancreatin (CAS number 8049-47-6) from porcine pancreas was obtained from Sigma-Aldrich (Oakville, ON, Canada). Bromocresol Green (B-6771) for the pH test, perhydrol (30% H_2_O_2_), Bradford reagent for the protein assay, and bovine serum albumin (CAS 9048-46-8) were all supplied by Sigma Aldrich (St. Louis, MO, USA). Eudragit EPO was purchased from Evonik industries (Darmstadt, Germany). Commercial HPMC capsules containing catalase (Go Away Gray 10,000) were purchased from Rise-N-Shine (Sparta, NJ., USA), and “Delayed release (DR)” capsules containing catalase (Catalase Antioxidant Caps) were from Suzy Cohen (Scottsdale, AZ, USA).

Simulated gastric fluid (SGF) and simulated intestinal fluid (SIF) were prepared according to US Pharmacopeia guidelines. In some experiments, 10 g of pancreatin were added to SIF, and the pH was adjusted to 6.8 in a final volume of 1000 mL. Acetate buffer was prepared by dissolving 2.99 g of sodium acetate in water and adding 14 mL of glacial acetic acid. The solution was diluted to 1000 mL, with the water adjusting pH to 4.5. The solution of phosphate-buffered saline pH 7.2 was prepared starting with 800-mL distilled water where 8 g of NaCl, 0.2 g of KCl, 1.44 g of Na_2_HPO_4_, and 0.24 g of KH_2_PO_4_ were dissolved. The pH was adjusted to pH 7.2 with HCl, and the volume was completed to 1 L.

### 2.2. Methods

#### 2.2.1. Synthesis of Starch Derivatives

**Figure 1:**
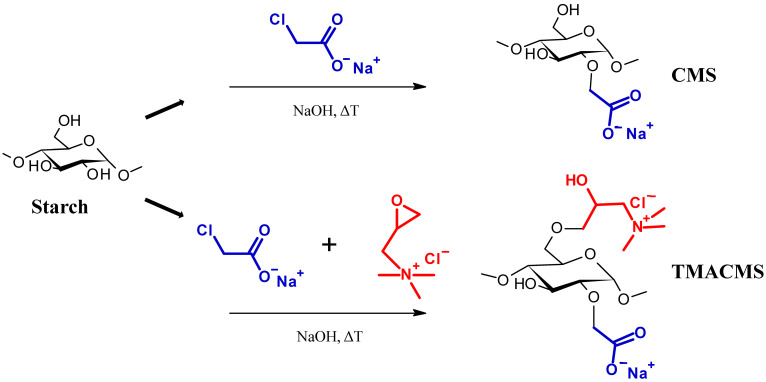
Schematic presentation of the synthesis of the CarboxyMethylStarch (CMS) and TriMethylAmineCarboxy. MethylStarch (TMACMS) excipients.

Two starch-based excipients were synthetized, as shown in Figure 1. The first excipient, CarboxyMethylStarch (CMS), is an anionic modified starch derivative carrying carboxylic groups (–COO^−^). The second excipient, TriMethylAmmoniumCarboxyMethylStarch (TMACMS), is an ampholytic starch carrying carboxylic and quaternary amine groups, –N^+^(CH_3_)_3_. These polymeric excipients were prepared separately following the method described by Sakeer et al. [24]. To synthesize CMS, 25 g of native starch (Hylon VII) were suspended in 200 mL of distilled water and then mixed with 300 mL of 5M NaOH for gelatinization, continuing the stirring and maintaining the pH (about 9) for 1 h at 60 °C. Then, 18.75 g of SMCA rapidly dissolved in water were added under stirring, and the reaction was continued under mild stirring at 60 °C for 1 h. After that, the solution was cooled to 4 °C and the pH adjusted to 7 with glacial acetic acid. The obtained CMS was finally precipitated and washed with methanol: distilled water (60:40 *v/v*) several times until a conductivity lower than 100 µS/cm was reached.

The final drying was done under vacuum and using pure acetone. The CMS powder was sieved to retain only the particles smaller than 300 µm. Ampholytic starch TMACMS was prepared as previously described by Sakeer et al. [24], with the mention that amounts of 18.74 g SMCA and of 18.74 g GTMAC were simultaneously added to gelatinized starch. When the solution was cooled down, the pH was decreased to 5 with glacial acetic acid. Precipitation, washing, and drying were done using the same method as explained above for CMS to obtain a TMACMS powder. All syntheses were done in triplicate, and the obtained powders were mixed to generate a homogeneous product that was subsequently used for characterization and all other tests.

#### 2.2.2. Characterization of Polymeric Powders

The new prepared excipients were characterized in order to confirm the presence of ionic groups and determinate the water content.

#### 2.2.3. Degree of Substitution (DS)

The degree of substitution of -OH by a carboxymethyl group (DS_COO^−^_) grafted on CMS or on TMACMS was measured using the back titration method [28]. An amount of 200 mg of the CMS or TMACMS was solubilized in 20 mL of NAOH (0.05 M), and a solution of HCl (0.05 M) was used to titrate the excess of NaOH that did not react with carboxylic groups. The equivalence point was observed with a phenolphthalein indicator. The titration of 20 mL of NaOH (0.05 M) served as a blank. All titrations were done in triplicate. The DS_COO^−^_ was calculated according to the following formula:

$$X=\left(V_{b}-\;V_{s}\right)\;\times C_{\mathrm{HCl}}$$

(1) $${\mathrm{DS}}_{{\mathrm{COO}}^{-}}=\;\frac{162X}{m-58X}$$

where $V_{b}$ and $V_{s}$ (L) are the titration volumes of HCl (0.05 M) for the blank and the samples, respectively. C_HCl_ is the concentration of HCl (mol/L), 162 (g/mol) is the molecular weight of one glucose unit, 58 (g/mol) is the increase in the weight of one glucose unit after substitution of a hydroxyl group by a carboxymethyl group, and m (g) is the mass of dry powders.

#### 2.2.4. Fourier-Transform Infrared (FTIR)

FTIR spectra of the native starch (Hylon VII), CMS, and TMACMS powders were recorded by a Thermo Scientific Nicolet 6700/Smart iTR (Madison, WI, USA) using a diamond crystal with 64 scans/min at 4-cm^−1^ resolution in the spectral region 4000–500 cm^−1^.

#### 2.2.5. X-ray Diffraction

A Bruker D8 Advance device (Munich, Germany) was used to measure the X-ray diffraction of the obtained powders and to evaluate their morphology. CuKα radiation at a wavelength of 1.5406 Å was used in reflectance mode, and a scanning rate of 0.05°/min in the 2θ range of 5–50° was applied on all samples. The X-ray diffraction spectra were analyzed with OXSAS™ software.

#### 2.2.6. Preparation of Tablets and Their Characterization in the Dry Phase

Monolithic tablets of 500 mg with 30% or 50% (*w*/*w*) of CAT were prepared by mixing the matrix-forming excipient with 150 mg or 250 mg of CAT powder, respectively. The excipients were initially mixed manually with a spatula and then transferred into a 25 mL plastic bottle and mixed for 10 min in a rotary device at 50 rpm. Monolithic tablets of 300 mg with 30% (*w*/*w*) of an active agent were prepared by mixing the matrix-forming excipient with 90 mg of CAT powder.

In order to visualize the behavior of tablets when they are immersed in media at various pH, a pH indicator was incorporated into each tablet. The mixture consisted in 69% excipient (345 mg), 30% (*w*/*w*) CAT (150 mg), and 1% (*w*/*w*) bromocresol (5 mg). The tablets containing Eudragit EPO were prepared with 30% (*w*/*w*) CAT; 55% (*w*/*w*) of the polymeric excipient (CMC, CMS, or TMACMS); and 15% (*w*/*w*) Eudragit. The excipients were initially mixed manually with a spatula and then transferred into a 25 mL plastic bottle and mixed for 10 min in a rotary device at 50 rpm.

The obtained mixtures were submitted to direct compression at 2.5 T/cm^2^ to obtain monolithic tablets. Flat-faced punches with a diameter of 9.5 or 13 mm were used on a Carver hydraulic press Model C 3912 Hydraulic Cylinder (Wabash, IN, USA).

Tablets of 100% (*w*/*w*) CAT were prepared by direct compression of 500-mg CAT powder.

The dimeter and thickness of all dry tablets were measured, and their hardness was tested using a tablet hardness tester Erweka model TBH 125 series (ERWEKA GmbH, Langen, Germany). The dimensions and hardness measurements were done in triplicate.

#### 2.2.7. Determination of the Fluid Uptake and Erosion

A study of fluid uptake and erosion properties was carried out on tablets of 500 mg containing 30% (*w/w*) CAT. Three dry tablets of each formulation were weighed (W_1_) and immersed in 50 mL of SGF for 2 h and then transferred to SIF. At different time intervals, the tablets were withdrawn from SIF and carefully weighed (W_2_). The recovered tablets were then dried at 40 °C for 48 h to obtain a constant weight (W_3_). Fluid uptake was calculated as follows:

(2) $$\%\;\mathrm{Weight}\;\mathrm{change}=\;\frac{W_{2}-W_{1}}{W_{1}}\times 100$$

The degree of erosion of tablets was determined according to the following equation:

(3) $$\%\;\mathrm{Erosion}=\;\frac{W_{1}-W_{3}}{W_{1}}\times 100$$

#### 2.2.8. In Vitro Dissolution Tests

Preliminary disintegration tests were carried out on 300-mg and 500-mg tablets (Table 1). Three tablets of each formulation were incubated in 50-mL SGF for 1 h and then transferred to 50-mL SIF at 37 °C and 50 rpm to be evaluated visually at every hour to determine the duration of their mechanical integrity. The tests were performed using an incubator shaker (series 25D New Brunswick Scientific Co., Edison, NJ, USA).

In vitro drug release tests were carried out by incubating the CAT tablets in 50-mL SGF at 50 rpm for 1 h or 2 h and then transferred to 50-mL SIF. All tests were done in triplicate at 37 °C and 50 rpm monitoring the enzymatic activity of CAT at a predetermined time interval (1 h) and for a period up to 8 h. Samples of 1 mL were withdrawn, filtered, and the enzymatic activity measured as described below.

#### 2.2.9. Catalase Activity

The enzymatic activity of the catalase was determined spectrophotometrically (Beckman DU-6, Beckman Coulter, Mississauga, ON, Canada) by monitoring at 240 nm the H_2_O_2_ decrease during catalysis [29]. The unit of enzymatic activity (U) is defined as the amount of the enzyme that catalyzes the conversion of one micromole of substrate per minute under the specified conditions of the assay method. A calibration curve was initially obtained using hydrogen peroxide 30% *w*/*w* (Sigma-Aldrich, H1009) diluted with 0.5 M phosphate-buffered saline (PBS), pH 7.2, and the measurements were done at 240 nm and thermostated at 25 °C. For the enzymatic test, 50 µL of the catalase sample were placed in a 1.5 mL quartz cell and mixed quickly with 1.45 mL of peroxide 0.03% The absorbance at λ 240 nm was recorded at time 0 and after one minute. For each sample, the dosage was done in triplicate.

#### 2.2.10. Protein Dosage

Protein concentrations of CAT were determined by the Bradford assay [30] using bovine serum albumin as the standard and monitoring the absorption at 595 nm.

## 3. Results and Discussion

### 3.1. Characterization of Excipients

**Figure 2:**
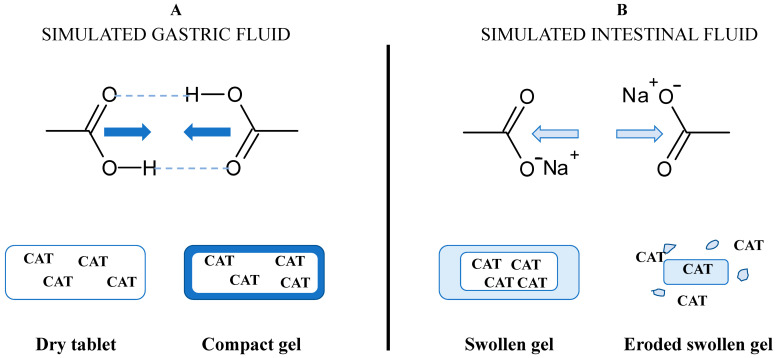
Concept of the formulation design of catalase (CAT) using excipients carrying carboxyl groups and the hypothetical mechanism of gastroprotection in (**A**) simulated gastric fluid (SGF) and of controlled release in (**B**) simulated intestinal fluid (SIF).

The degree of substitution (DS) for the carboxymethyl groups represents the average number of hydroxyl groups carrying –CH_2_-COO^−^ anionic functional groups (DS_COO^−^_) per glucose unit. The DS_COO^−^_ measured by back titration using Equation (1) were 0.08 for CMS and 0.03 for TMACMS, respectively. The presence of anionic carboxylic groups on starch derivatives is important for the stabilization of polymeric matrices and for the gastroprotection of CAT. The excipients carrying anionic groups will become protonated in an acidic medium (Figure 2), producing a compact outer gel around the tablets. The anionic monolithic matrices will better prevent SGF penetration and protect CAT during the tablets passing through the stomach. When the medium becomes neutral (SIF), the carboxylic groups will be deprotonated, and under a salt form (–COO^−^Na^+^), they will be able to hydrate and swell [31]. The medium penetration is accelerated, and the tablet texture changes continuously, becoming a swollen gel that finally erodes and releases CAT.

**Figure 3:**
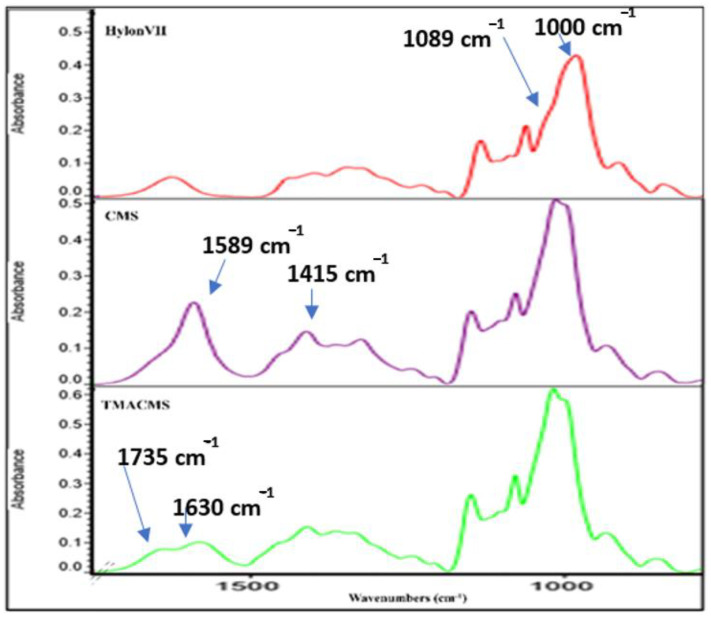
Fourier-transform infrared (FTIR) spectra of native starch (Hylon VII) and of the CMS and TMACMS derivatives.

Fourier-transform infrared (FTIR) spectra also gave information on the extension of starch modification with carboxylic groups (for CMS) or either carboxylic and amine groups (for TMACMS). The FTIR spectra of the native starch and of its derivatives show bands at 3331 cm^−1^ and 2980 cm^−1^ that correspond to -OH and -CH-stretching vibrations (Figure 3).

We focused our interpretation on the bands at 1089 and 1000 cm^−1^ associated with –CH_2_-O-CH_2_-stretching vibration [32,33]. The FTIR of CMS show two specific bands at 1589 and 1415 cm^−1^ that represent the stretching vibration of carboxylate –COO^−^ [33]. As for the TMACMS spectrum, it shows a shoulder of the band at 1589 cm^−1^ corresponding to the stretching vibration of –COO^−^ slightly decreased due to the grafting of the two ionic groups on starch. It also shows a new band between 1630 and 1735 cm^−1^ that represents the vibrations of the N^+^(CH_3_)_3_ group. This confirmed the successful synthesis of ampholytic starch. For TMACMS, the DS of the hydroxyl groups with the trimethyl amine groups was 0.055 and was calculated by elemental analysis. The results were given by the percentage of molecular mass for each element.

**Figure 4:**
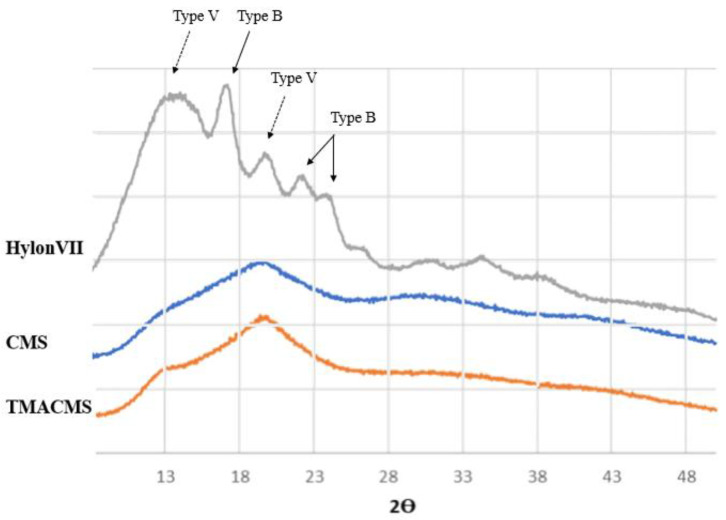
X-ray diffraction patterns of Hylon VII, CMS, and TMACMS. The diffractograms of CMS and TMACMS exhibit larger less-ordered zones (V-type), whereas the more crystalline zones (B-type) are decreased.

The powders of the native starch and of its derivatives were also analyzed by X-ray diffraction (Figure 4). The native starch has a more organized semicrystalline structure compared to its derivatives. Specific bands at 17.09°, 22.13°, 23.52°, and 24.08° Bragg angle (2θ) showed the presence of double-helices (B-type), whereas diffractions at 12.9° and 19.89° were associated with the presence of the simple helical conformation (V-type).

The spectra of the CMS and of TMACMS show less organization than unmodified native starch. The modifications have an impact on the powder morphology: the peaks representing the more disorganized zones (V-type) are still present after derivatization, whereas the ones corresponding to more crystalline zones (B-type) are decreased [34]. This can be due to the grafting of the functional groups CM and TMA on the polysaccharide backbone by substitution of the hydroxyl group [24]. For starch materials, the hydroxylic groups play an important role by their involvement in hydrogen associations and in the stabilization of polysaccharidic chains that can adopt various conformations, depending on various parameters (i.e., starch origin, water molecules present in the structure, etc.) [35].

Chemical modifications operated by the introduction of ionic functional groups on polysaccharidic chains affected the chain conformations with the destabilization of hydrogen association and the loss of B-type (double-helix) morphology. These modifications can be captured by X-ray diffraction analysis and be linked to the capacity of starch derivatives to generate polymeric networks and hydrogels [36]. The semicrystalline pattern of both derivatives is a key factor in the preparation of monolithic tablets designed for controlled drug release.

The slightly more organized structure of ampholytic starch can be explained by a new organization of polymer chains due to possible enhanced ionic interactions between the negatively charged COO^−^ groups and positively charged trimethylammonium functions of TMACMS. These ionic interactions are responsible for matrix self-stabilization having an impact on controlled CAT release [37].

### 3.2. Formulation Studies

Tablets with CAT as a bioactive principle and various excipients (CMC, CMS, or TMACMS) were prepared, and their physical characteristics are summarized in Table 1. The formulations based on the starch derivatives generated very hard tablets, all of them exhibiting values overpassing 150 N compared with CMC-based tablets having around 100 N.

Tablet behaviors in terms of mechanical resistance in various media were followed by their exposure to 50 mL of SGF for 1 h and then transferred to 50 mL of SIF (incubated in a shaker at 50 rpm, 37 °C). The tablets of TMACMS + 30% catalase (*w*/*w*) resisted longer in SIF (more than 5 h), presenting higher hydration and disintegration times (Table 1). Both tablets of 300 mg and 500 mg showed good values of hardness and disintegration times in SIF, especially for the TMACMS + 30% (*w*/*w*) catalase formulation. Since no major differences in these features were found between tablets of 300 mg and of 500 mg, the rest of the studies were carried out on 500 mg tablets, aiming to incorporate more catalase.

### 3.3. Gastroprotection

**Figure 5:**
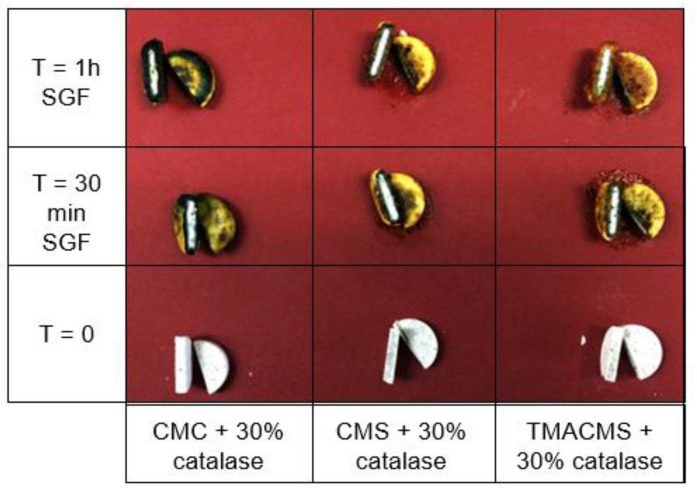
Cross-sectional cuts of the tablets containing a pH indicator and incubated in SGF. CMC: CarboxyMethylCellulose.

Tablets based on CMC, CMS, and TMACMS and containing a pH indicator (Bromocresol) showed a change of color on the surface due to the acidity of the simulated gastric fluid, whereas the inside cores remained dry and unchanged in color (Figure 5). This could be interpreted as a good protection of the enzyme against the acidity of the stomach.

### 3.4. Fluid Uptake, Swelling, and Erosion

**Figure 6:**
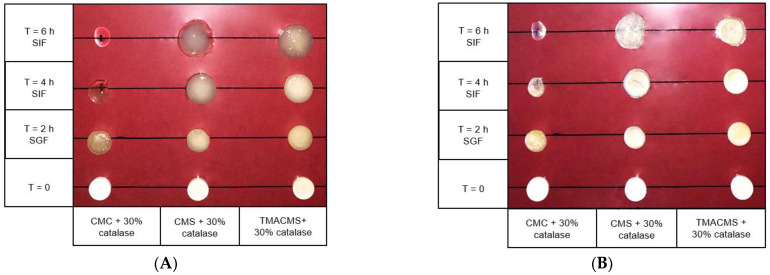
Pictures of the CMC, CMS, and TMACMS tablets containing 30% catalase at T = 0 (dry) and T = 2 h in SGF, followed by T = 4 and 6 h in SIF. Pictures were taken on wet (**A**) and on same tablets dried 48 h at 50 °C (**B**).

**Figure 7:**
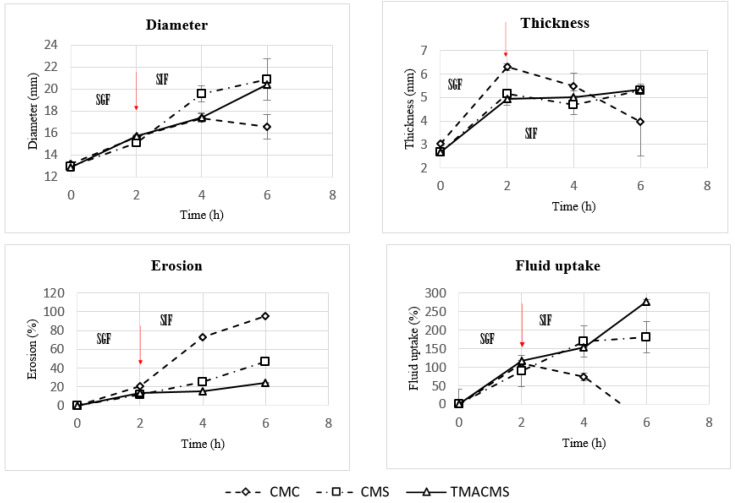
Diameter, thickness, erosion, and fluid uptake changes of tablets of CMC, CMS, and TMACMS tablets containing 30% catalase. Tablets were incubated in SGF for 2 h and, then, were transferred to SIF at T = 2 h. The arrows mark the transitions from SGF to SIF.

Tablets of 500 mg with 30% catalase were exposed to SGF for 2 h at 50 rpm and then transferred to the SIF for 2 and 4 h. They were weighed at the initial T = 0 h, then at T = 2 h, T = 4 h, and T = 6 h and, after drying at 50 °C for 48 h, at T = 0 h, T = 2 h, and T = 4 h. Additionally, their integrity, weight, diameter, and thickness were measured at T_0h_, T_2h_, T_4h_, and T_6h_ (results shown in Figure 6 and Figure 7).

The CMC-based tablets exhibited a low fluid uptake after 2 h in SGF that changed when transferred to SIF. Additionally, a strong erosion profile was found: 100% in SIF. Differently, the CMS-based tablets showed a limited erosion of 18% after 2 h in SGF and of 50% after 4 h in SIF, along with a low fluid absorption. For the tablets of TMACMS, an even lesser erosion (20%) after 4 h in SIF was found, but they showed a stronger fluid uptake behavior, reaching 250% after 4 h in SIF. It is worth noting that the behavior of these tablets with ampholytic starch was not affected by the change of pH when transferred from SGF to SIF. Furthermore, the TMACMS tablets with 30% catalase exhibited a good stability in SGF and SIF: the increase of both weight and shape was stable and proportional to the fluid uptake. This behavior could be due to the strong interactions between the cationic and anionic groups present on the ampholytic starch chain as result of its derivatization.

### 3.5. In Vitro Dissolution Assays

**Figure 8:**
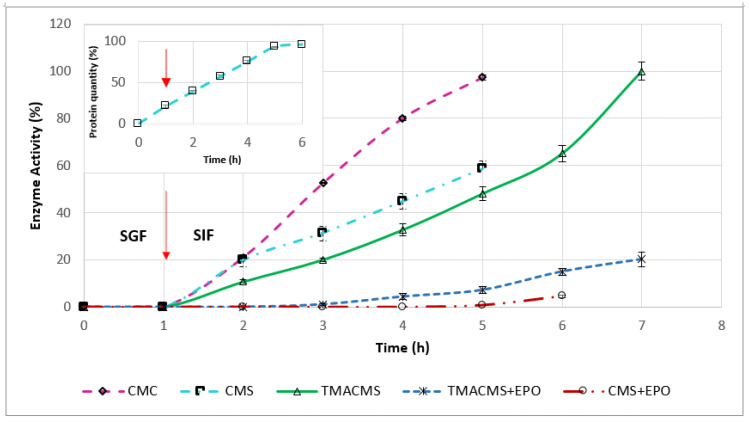
Release profile of catalase from the tablets with CMC, CMS, TMACMS, CMS + EPO, and TMACMS + EPO as excipients all containing 30% catalase (*n* = 3). Tablets of each formulation were incubated separately in 50 mL of SGF (37 °C, 50 rpm). After 1 h, the tablets were transferred to 50 mL of SIF, and the activity of catalase was measured every hour. The arrow marks the transition of the tablets from SGF to SIF. Insert: Release profile of the protein quantity from the tablets of CMS containing 30% catalase.

Monolithic tablets based on different excipients and containing 30% CAT were exposed to SGF for 1 h, then transferred to SIF and followed until complete disintegration. Samples taken every hour to measure the CAT activity generated the release profiles shown in Figure 8. The enzymatic activity was preserved by all three formulations. CMC and CMS prolonged the CAT release up to 5 h. CMC preserved 100% of the enzymatic activity, whereas CMS provided less protection, and 40% of the activity was lost.

The ampholytic TMACMS matrices showed the longest time to maintain the tablet shapes (for up to 7 h) and provided 100% release of the CAT (markedly better than that obtained with the anionic CMS and CMC excipients). This behavior could be related to the electrostatic interactions between the anionic –COO^−^ and cationic –N^+^(CH_3_)_3_ groups that can act as a supplementary stabilization factor of the polymeric matrix. Some interactions could be also established between the ionic groups of certain amino acids (aspartate, glutamate, and lysine) present in the catalase structure and those of the ampholytic matrix chains.

A structural reorganization affecting the physical stabilization is another possibility that could generate a more compact matrix, a better control of swelling and, implicitly, of the enzyme release. In fact, the longer time of drug release (Figure 8) seems related to the swelling dynamics of the TMACMS tablet that exhibited the highest fluid uptake associated with a low erosion rate (Figure 7). Although the CMS tablets were completely disintegrated, the released enzymatic activity did not reach 100% of the total activity, which probably means that the CMS polymer did not form an outer gel compact enough to prevent a partial enzyme inactivation or leaking in SGF. The pH has an influence on the ionization of amino acids contained in CAT composition. In SGF, at an acidic pH, the state of ionization of the amino acids (carrying carboxyl or amine functional groups in their side chains) is changed due to a higher concentration of H^+^ ions. In these conditions, the hydrogen bonds that determine the 3D shape of the protein are altered, conducting a loss of activity (denaturation) of CAT.

To test this hypothesis, the amount of protein released was measured every hour for a 6-h interval. The profile in Figure 8 (insert) showed a constant release in SGF and in SIF. This suggests a kind of limited loss of enzyme from the surface (burst effect) or from the outer layers of the tablets during one-hour immersion in SGF.

The same tests performed on tablets of 100% CAT showed a fast and complete disintegration in SGF (in few minutes). When the CAT tablets were directly submerged in SIF, they disintegrated after 1 h. These results reflect that the enzymes need optimal pH values to act as efficient catalysts. Outside optimal pH values, the global charge of the enzyme will be altered, inducing changes in the protein solubility and of the overall shape. When free CAT tablets were exposed to the acidic pH of SGF, this change in the shape of the active site diminishes the CAT ability to bind to the substrate, thus conducting its inactivation.

Tablets of 50% CAT with CMC, CMS, and TMACMS were also tested, and they did not preserve the enzymatic activity of the catalase. This also confirms that the key factor in the controlled release of CAT is the compact matrix formed by the polymeric excipient protecting the enzyme.

To gain more insights on the mechanisms involved in controlled catalase release and the role of electrostatic interactions, a synthetic polymer, methacrylate co-polymer Eudragit EPO, carrying cationic (tertiary amine) groups was used at 15% in combination with CMS or with TMACMS to prepare monolithic tablets (keeping the same 30% CAT loading). This formulation was aimed to elucidate if more cationic groups would have an impact on the behavior of matrices and on the CAT release. Eudragit EPO is a currently used excipient in oral dosage forms [38]. The tablets were exposed to similar conditions: 1 h in SGF at 50 rpm, 37 °C, and then transferred to SIF with follow-up until complete disintegration. The profiles (Figure 8) showed that CMS tablets with Eudragit EPO released 5% of the total enzymatic activity after complete disintegration in SIF. Similarly, tablets of TMACMS with Eudragit EPO ensured the release of only 20% of the enzymatic activity in SIF (80% were lost) compared to the dissolution profiles from tablets without Eudragit EPO. All monolithic tablets containing methacrylate eroded and did not exhibit significant swelling. Maybe the presence of cationic groups [39] on methacrylate chains could favor the formation of complexes with the carboxylic groups of CMS and of TMACMS excipients and hinder the matrix hydration. In the light of these findings, it seems that the CAT release mechanism from the proposed matrices is mainly driven by swelling.

**Figure 9:**
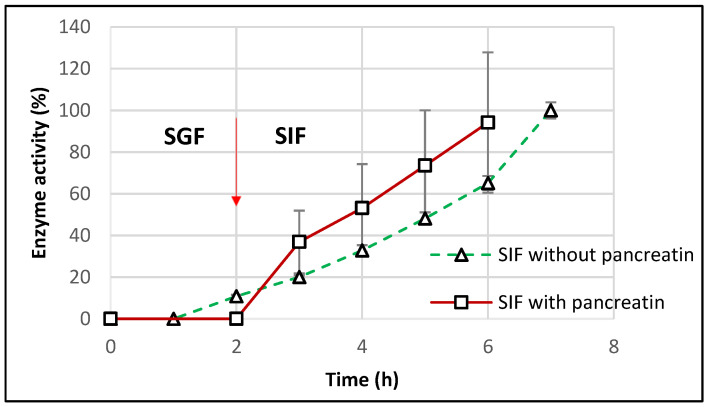
Release profile of catalase from tablets of TMACMS containing 30% CAT in SGF to SIF with and without pancreatin. The arrow marks the transition of the tablets from SGF to SIF.

For a closer similarity with the in vivo conditions, the dissolution tests were repeated for the optimal starch-based formulations in SIF containing pancreatin (as per United State Pharmacopeia guidelines). After incubation for 2 h in SGF, TMACMS tablets were transferred to SIF medium with pancreatin, and the results (Figure 9) showed a controlled release of CAT with a linear profile.

The catalase activity reached 100% after 6 h (2 h in SGF + 4 h in SIF), showing a good protection of catalase against the proteolytic enzymes of pancreatin. However, the results showed more variation that could be explained by the presence of pancreatin, since the method of measuring the catalase activity, although precise, remains sensitive [40]. The effect of pancreatin was followed only for the optimal formulation with starch-based excipients to understand if amylase could drastically affect the matrix features due to a starch susceptibility to amylolysis. The aspect of excipient-free catalase was approached in a previous study [21], clearly showing an almost complete loss of the catalase activity in SIF with pancreatin. This result supports the essential protective role of the excipients of CAT activity in the simulated intestinal medium.

### 3.6. Commercial Products

Two commercial catalase supplements in capsule form were tested in the same conditions: HPMC capsules (Rise-N-Shine) and DR™ acid-resistant capsules (Suzy Cohen). In SGF, both of them were completely dissolved, and they lost their activity after 1 h. When exposed only to SIF, the DR capsules showed no measurable catalase activity, whereas the HPMC capsules released 100% of their labeled catalase activity in 2 h. These results confirm that HMPC or DR capsules do not offer the same level of gastroprotection as monolithic matrices like TMACMS.

**Figure 10:**
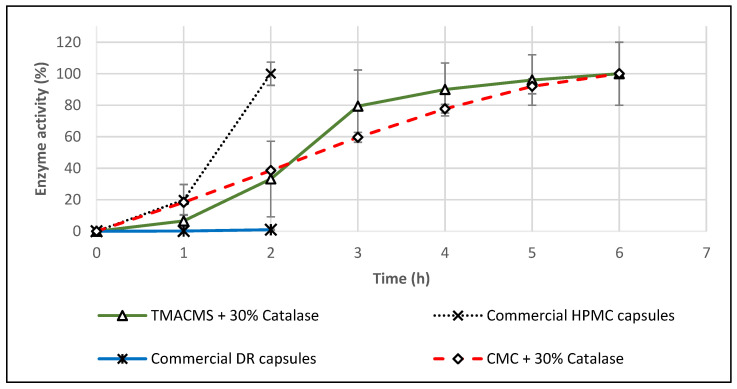
Release profiles of catalase from tablets based on CMC and TMACMS and from hydroxypropyl methylcellulose (HPMC) and delayed release (DR) capsules. All tests were performed in triplicate in acetate buffer, pH 4.5, at 37 °C, 50 rpm.

Considering that, on commercial product flyers, it is suggested to take one capsule daily with food, additional in vitro tests were conducted also in acetate buffer at pH 4.5 (pH corresponding to the simulated fed state). After one hour in this dissolution fluid, the HPMC capsules were completely disintegrated, and the content formed a few agglomerates that were totally dissolved in 2 h, although the CAT activity was recovered at 100% (Figure 10). The fact that the capsules dissolve in this dissolution medium suggests that the enzyme release cannot be controlled during the gastrointestinal transit. In the case of DR capsules, they were swollen after 1 h and disintegrated completely after 2 h. The results shown in Figure 10 indicate no detectable CAT activity for this product. Catalase–TMACMS tablets exhibited a release profile very similar to those performed in SGF/SIF media (Figure 8), indicating the capacity of this excipient to compensate for the variations of pH of various dissolution media.

## 4. Conclusions

This report proposes a new approach for the formulation of catalase to treat dysfunctions related to the excess of hydrogen peroxide observed in intestinal inflammation. By breaking down hydrogen peroxide, catalase could contribute to diminishing the damage at level of the intestinal mucosa. The formulation design was based on polymeric excipients carrying ionic groups that generated matrices able to protect CAT during passage through the stomach and modulate its release to the intestines. From the three ionic excipients (CMC, CMS, and TMACMS) evaluated as matrix-forming materials, the ampholytic TMACMS excipient provided the best release profile from tablets loaded with 30% CAT. Furthermore, the monolithic matrices based on TMACMS ensured an effective protection of the enzyme against the acidity of the gastric fluid, as well as against the pancreatin present in the intestinal fluid. It was found that the mechanism of CAT release was mainly based on the controlled hydration of the polymeric matrix, which was modulated by ionic groups grafted on the polysaccharidic chains. For the TMACMS, the simultaneous presence of anionic and cationic charges provided an enhanced matrix stability and a longer and targeted enzyme release compared with the other solid dosages under capsule form.

Considering the high patient-to-patient variability of the intestinal tract in terms of pH and residence time in different segments of the gastrointestinal tract, these findings suggest the ampholytic TMACMS excipient as a promising excipient for the formulation of sensitive bioactive agents (i.e., therapeutic enzymes). Its capacity to protect and to modulate the release of a biological active agent could be beneficial in irritable bowel syndrome and in IBD such as Crohn disease and ulcerative colitis.

## Figures and Tables

**Table 1:** Summary of the physical properties of tablets with 30% catalase (CAT) loading. The disintegration times were measured in 50 mL of simulated intestinal fluid (SIF) while incubated in a shaker at 37 °C after being initially incubated for 1 h in simulated gastric fluid (SGF). CMC: CarboxyMethylCellulose, CMS: CarboxyMethylStarch, and TMACMS: TriMethylAmineCarboxyMethylStarch.

	Formulation	CAT Load (% *w*/*w*)	Table Weight (mg)	Dimensions of Dry Tables		Hardness (N)	Time of Complete Disintegration in SIF
				Dimeter (mm)	Thickness (mm)		
1	CMC + CAT	30	300	9.65 ± 0.11	3.17 ± 0.07	103.8 ± 0.5	3–4 h
2	CMS + CAT	30	300	9.52 ± 0.12	2.91 ± 0.05	>150	3–4 h
3	TMACMS + CAT	30	300	9.63 ± 0.19	2.84 ± 0.08	>150	>5 h
4	CMC + CAT	30	500	13.21 ± 0.15	3.06 ± 0.06	99.6 ± 0.03	3–4 h
5	CMC + CAT	30	500	12.92 ± 0.17	2.63 ± 0.07	>150	3–4 h
6	TMACMS + CAT	30	500	12.93 ±0.15	2.67 ± 0.05	>150	>5 h

## Data Availability

The data presented in this study are available on request from the corresponding author. The data are not publicly available due to their possible use for other on-going studies.

## Abbreviations

**Abbr.**	**Expansion**
CAT	Catalase
CD	Crohn’s disease
CMC	Sodium CarboxyMethylCellulose
CMS	CarboxyMethylStarch
DS	Degree of substitution
DR	Delayed Release
FTIR	Fourier-transform infrared
GTMAC	Glycidyltrimethylammonium
IBD	Inflammatory bowel disease
MIF	Migration inhibitory factor
SGF	Simulated gastric fluid
SIF	Simulated intestinal fluid
SMCA	Sodium monochloroacetate
TGA	Thermogravimetric analyses
TMACMS	TriMethylAmmoniumCarboxyMethylStarch
ROI	Reactive oxygen intermediates
UC	Ulcerative colitis
